# 659. Correlate Clinically and More-Use of Interpretative Comments in Clinical Microbiology Reporting

**DOI:** 10.1093/ofid/ofab466.856

**Published:** 2021-12-04

**Authors:** Raksha Kochhi

**Affiliations:** St Martha’s Hospital, Bangalore, Karnataka, India

## Abstract

**Background:**

Microbial identification & antibiotic susceptibility testing is an important investigation in clinical microbiology laboratory. In many centres in India the report has only the isolate and antibiotics tested. The additional comments if added give guidance to the clinicians to utilize the results. Pre-analytical issues of adequate & relevant clinical history, appropriate sampling techniques, timely transport & storage, history of antibiotic usage along with post analytical issues of recommended line of antibiotic therapy and infection control practices are better addressed with this practice.

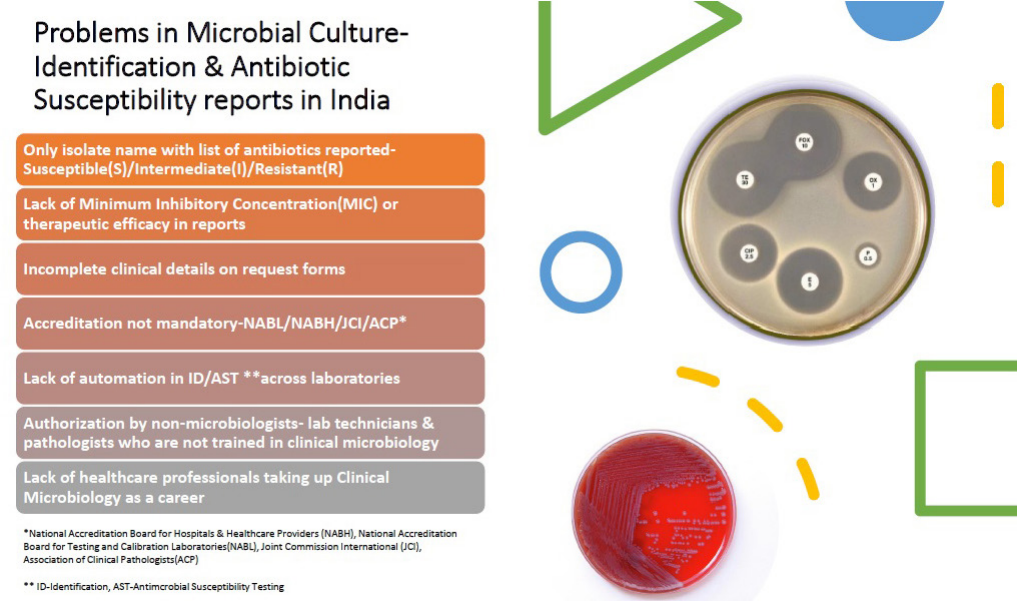

**Methods:**

This was a prospective qualitative study from the period of January 2017-March 2021 where in the standard operating protocol of Clinical Microbiology was reviewed and appropriate comments were included in the Laboratory Information System once the isolate was identified using VITEK 2, automated ID/AST instrument and interfaced. The Clinical Microbiologist would then review the comments upon discussion with the clinicians and then authorize reports. The reports included sample & isolate specific details , recommended antibiotic therapy and infection control related comments. This was based on standard international and national guidelines (CLSI, EUCAST, IDSA, IAP, and National Treatment Guidelines of India).

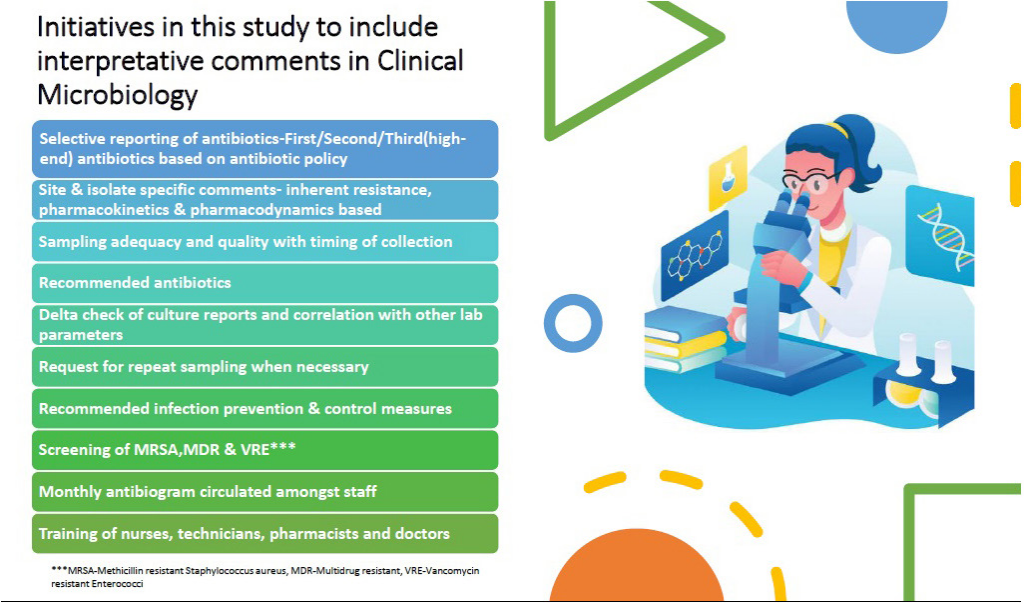

**Results:**

There was a gradual improvement in completion of request forms with clinical history, sample site and antibiotic history being mentioned. This was assessed through periodic audits conducted every quarter from 36% in March 2017 to 95% in March 2021. Clinical communication with the microbiology laboratory also showed improvement with documentation. Feedback from clinicians was also taken on the utility of these comments, (87/120)72.5% of the clinicians found them useful(Grade 5). (32/120) 26 %(Grade 3) of the clinicians had concerns about the turnaround time and requested for provisional reports.

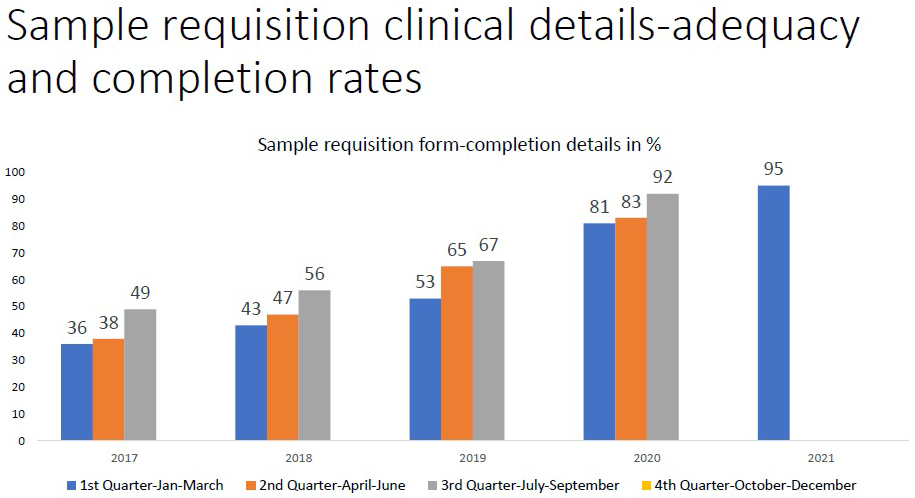

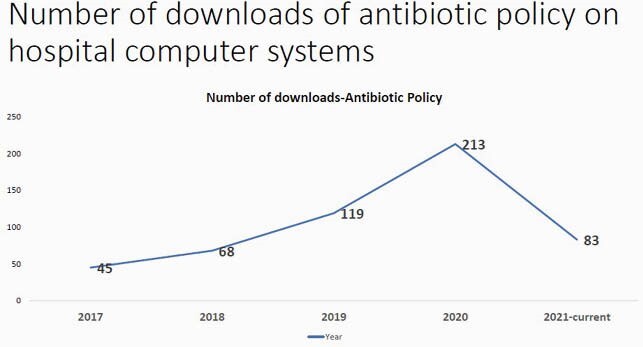

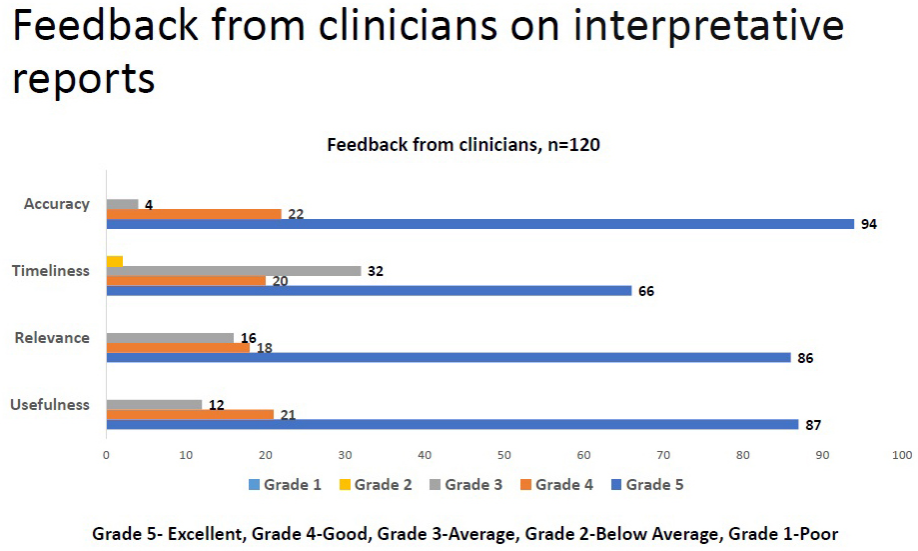

**Conclusion:**

Interpretative comments in reports act as a bridge between clinical microbiology, infectious diseases and infection control. They help us to choose the correct antibiotics or sometimes no antibiotics when the situation demands it. With all the recent advancements, the clinico-microbiological utility of culture reports is the need of the hour.

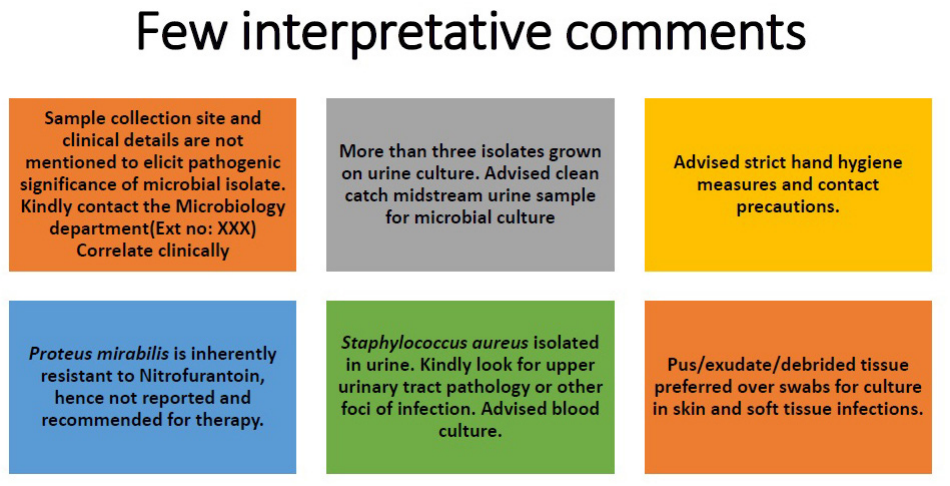

**Disclosures:**

**All Authors**: No reported disclosures

